# Establishment and evaluation of a novel tool based on inflammation-nutrition derived biomarkers for early diagnosis of diabetic foot ulcers

**DOI:** 10.3389/fimmu.2026.1794011

**Published:** 2026-03-17

**Authors:** Jie Wang, Yan Zhang, Qiong Wang, Jianbo Sun, Yinan Jin, Hongmou Zhao

**Affiliations:** 1 Department of Foot and Ankle Surgery, Honghui Hospital of Xi’an Jiaotong University, Xi’an, Shaanxi, China; 2 Diabetic Foot Multidisciplinary Team (MDT) Center, Honghui Hospital of Xi’an Jiaotong University, Xi’an, Shaanxi, China

**Keywords:** diabetic foot ulcers, neutrophil percentage-to-albumin ratio, nomogram, personalized medicine, prediction model

## Abstract

**Objective:**

The study aimed to investigate the relationship between neutrophil percentage-to-albumin ratio (NPAR) and diabetic foot ulcer (DFU) in Chinese adults, further establish a clinical predictive model, and verify its effectiveness.

**Methods:**

We retrospectively collected and analyzed clinical data from a total of 1,002 diabetic patients at Honghui Hospital of Xi’an Jiaotong University between January 2024 and January 2026. The association between the NPAR and DFU risk was assessed using a logistic regression. Moreover, the nonlinear relationship was further characterized through smooth curve fitting and generalized additive model analysis. The predictors were identified via the least absolute shrinkage and selection operator and multivariate logistic analysis. The discrimination and calibration of the nomogram were validated by receiver operating characteristic (ROC) curve and calibration curve. Decision curve analysis (DCA) was used to evaluate clinical usefulness and net benefits of the prediction model.

**Results:**

The multivariate logistic regression analysis demonstrated that NPAR (odds ratio [OR] =1.303, 95% confidence intervals [CI]: 1.212–1.402), age (OR = 1.058, 95% CI: 1.032–1.083), sex (female vs. male, OR = 0.475, 95% CI: 0.281–0.802), body mass index (20–25 kg/m² vs. <20 kg/m², OR = 0.184, 95% CI: 0.094–0.359; ≥25 kg/m² vs. <20 kg/m², OR = 0.445, 95% CI: 0.252–0.788), smoke (yes vs. no, OR = 1.735, 95% CI: 1.023–2.941), peripheral vascular disease (yes vs. no, OR = 5.522, 95% CI: 3.428–8.896), peripheral neuropathy (yes vs. no, OR = 6.914, 95% CI: 4.114–11.618), and hemoglobin (OR = 0.981, 95% CI: 0.967–0.996) were risk-associated indicators for DFU. The calibration curves for the training and validation cohorts both revealed good agreement. In addition, the area under the ROC curve values in the training and validation cohorts were 0.892 (95% CI: 0.864–0.919) and 0.877 (95% CI: 0.831–0.922), respectively, indicating good predictive discrimination. The DCA showed that the nomogram could provide clinical usefulness and net benefit.

**Conclusion:**

This study indicated a positive relationship between DFU risk and the integrated inflammatory-nutritional status represented by NPAR in the Chinese diabetic population. The DFU prediction model incorporating NPAR was validated for its effectiveness and clinical utility, providing evidence for the potential of NPAR as a risk-associated indicator measured at DFU diagnosis.

## 1 Introduction

Diabetic foot ulcer (DFU) is one of the most common and severe complications of diabetes. Meanwhile, its prevalence is increasing at an alarming rate, with 19%–34% of diabetic patients experiencing DFU in their lifetime (1). Approximately 20% of patients with DFU require hospitalization, and among hospitalized patients, 15% to 20% undergo lower extremity amputation. Even after initial ulcer healing, the recurrence rate of DFU is about 40% within one year and as high as 60% within three years (1, 2). In China alone, the cost of DFU management is projected to rise to $7.4 billion by 2030 (3). This imposes a heavy burden on individuals and the healthcare system. Studies have shown that the pathogenesis of DFU is closely associated with peripheral vascular and neuropathic disorders, abnormally elevated plantar pressure due to various causes, as well as systemic inflammation and nutritional status (4). Characterized by oxidative stress, hyperglycemia, ischemia, hypoxia, and persistent infection, DFU wounds pose significant challenges in clinical management (2, 5). Currently, the efficacy of standard treatments, primarily involving debridement, off-loading, and glycemic control, remains relatively limited. Particularly for many patients undergoing repeated amputations, postoperative satisfaction is often poor (2, 6, 7). With the recent advancements in preventive medicine, many studies have advocated for early screening of high-risk DFU populations and the implementation of targeted intensive prevention and individualized interventions. In particular, for patients in the early stage of DFU who have not yet exhibited clinical symptoms, early screening is of great significance. Research has indicated that effective risk assessment and management could prevent over 50% of DFU cases and related amputations (8, 9).

In recent years, numerous studies have reported that inflammation and nutrition-related biomarkers, including procalcitonin, C-reactive protein (CRP), erythrocyte sedimentation rate, interleukins, pentraxin-3, tumor necrosis factor-alpha, white blood cell count, and serum albumin, are emerging as useful references for the diagnosis of DFU (10, 11). However, single predictors may lack specificity and have limitations. DFU patients are in a chronic inflammatory state over the long term, which can lead to malnutrition through mechanisms such as restricted dietary quality and chronic inflammatory consumption (2, 5). Concurrently, nutritional deficiencies further prolong the inflammatory phase, impede collagen synthesis and wound healing, and worsen clinical outcomes (12, 13). Thus, inflammation and nutritional deficiency exacerbate each other in DFU patients. In contrast, a comprehensive biomarker that simultaneously reflects both inflammatory and nutritional statuses may be more holistic and effective for the early diagnosis of DFU. As is well known, neutrophils account for the highest proportion of human leukocytes and are closely associated with inflammatory processes and immune responses (14). Serum albumin is a key marker of nutritional health and also possesses antioxidant and anti-inflammatory properties (15). The neutrophil percentage-to-albumin ratio (NPAR), as a novel and effective biomarker, combines neutrophil percentage and albumin values to reflect systemic inflammation and nutritional status. This parameter can be calculated from routine blood tests and standard biochemical analyses, offering advantages such as easy accessibility, low cost, and high generalizability. Recent studies have linked NPAR to nonalcoholic fatty liver disease (16), osteoporosis in rheumatoid arthritis (17), depression (18, 19), asthma (20), psoriasis (21), periodontitis (22), Parkinson’s disease (23), as well as the diagnosis and prognosis of various cancers (24–29). However, no study has yet explored the relationship between DFU and the NPAR. Furthermore, although some studies have reported risk screening systems for DFU, related research remains exploratory and has not reached a unified consensus (30–33). How to predict the risk of DFU based on risk factors and develop effective preventive strategies remains an urgent issue to be resolved.

Against this backdrop, drawing on the latest advances in the prevention and management of DFU, the present study is designed to explore the potential correlation between DFU and NPAR. On this basis, a risk prediction model will be further constructed to quantitatively assess the likelihood of DFU onset among diabetic patients. It is anticipated that this model could serve as a straightforward, feasible, and easily scalable tool for the early identification of high-risk populations, which in turn will lay a solid foundation for the formulation and implementation of individualized patients management regimens.

## 2 Materials and methods

### 2.1 Study population

A retrospective collection and analysis of clinical datasets was performed on diabetic patients admitted to Honghui Hospital, Xi’an Jiaotong University, during the period from January 2024 to January 2026. Consecutive sampling was adopted to enroll patients in this study. Given that the amount of missing data in this study was far less than 5% of the total sample size, listwise deletion was adopted to handle the missing data. The study established the following inclusion criteria: (1) confirmation of type 2 diabetes mellitus in accordance with the diagnostic guidelines formulated by the World Health Organization; (2) attainment of the age of 18 years or above; (3) having complete clinical data. Conversely, the exclusion criteria were defined as follows: (1) a confirmed diagnosis of type 1 diabetes or secondary diabetes; (2) current pregnancy or lactation status; (3) diagnosis of thromboangiitis obliterans; (4) coexistence of malignant tumors, other severe infectious disorders, or cognitive dysfunction. This research protocol was implemented in strict compliance with the principles of the Declaration of Helsinki and had been formally approved from the Ethics Committee of Honghui Hospital Affiliated to Xi’an Jiaotong University (approval number: 2025-KY-211-01). As a retrospective cohort study, all clinical data pertaining to the enrolled patients were processed with anonymization to ensure confidentiality.

### 2.2 NPAR calculation and covariates

All data in this study were collected at the time of admission. Building on the findings of relevant prior studies and accumulated clinical practice experience, the present research screened and finalized a set of covariates. These included demographic indicators, including age, sex (male/female), body mass index (BMI), and residential area (urban/rural); lifestyle-related factors, including smoke status (yes/no) and alcohol consumption status (yes/no); comorbidity profiles, including hypertension, cardiovascular disease (CVD), peripheral vascular disease (PVD), and peripheral neuropathy (PN); as well as laboratory test parameters, such as hemoglobin, glycosylated hemoglobin, fasting blood glucose, lymphocyte count, uric acid, serum creatinine, and estimated glomerular filtration rate (eGFR) (34).

Albumin was measured in g/dL, the neutrophil percentage was measured as a proportion of the total white blood cell count, and the calculation formula for NPAR was as follows (35):


$$NPAR=\frac{Neutrophil\;percentage\;\%}{Serum\;albumin\;(g/dL)}$$

### 2.3 Statistical analysis

We first performed a normality test on all continuous variables using the Shapiro-Wilk test. For continuous variables that conformed to a normal distribution, the Student’s t-test was used to compare differences between the DFU and non-DFU groups; for continuous variables that did not conform to a normal distribution, the Wilcoxon rank-sum test was employed to compare intergroup differences. Homogeneity of variance was verified via the Levene’s test to ensure the applicability of parametric tests. In addition, continuous variables were expressed as mean ± standard deviation (SD), and categorical variables were described with constituent ratios. The association of NPAR with the risk of DFU was analyzed through logistic regression models, with corresponding odds ratio (OR) and 95% confidence intervals (CI) calculated. Smooth curve fitting (SCF) combined with generalized additive models (GAMs) were applied to explore the potential nonlinear relationships between the above indicators. Subgroup analyses stratified by age and sex were additionally conducted to verify the stratification effects of these two factors. To enhance analytical robustness and in line with field conventions (36–39), NPAR values were stratified into tertiles, with linear trend tests further conducted. The same analytical procedures were employed to investigate the correlation between NPAR tertiles (NPAR.T) and DFU risk. Three regression models were constructed with different covariate adjustment strategies: Model 1 was an unadjusted crude model; Model 2 was adjusted for age (when applicable), sex (when applicable) and BMI; Model 3 was a fully adjusted model incorporating age (when applicable), sex (when applicable), BMI, residential area, smoke, alcohol consumption status, hypertension, CVD, PVD, PN, hemoglobin, glycosylated hemoglobin, fasting blood glucose, lymphocyte count, uric acid, creatinine and eGFR. The predictive discriminative ability of NPAR for DFU was evaluated using the receiver operating characteristic (ROC) curve and the area under the curve (AUC), and the optimal cut-off value of NPAR for DFU identification was determined according to the maximum Youden index (sensitivity + specificity - 1). Moreover, the clinical application value of this cut-off value was further validated by calculating indicators including sensitivity, specificity, positive predictive value (PPV) and negative predictive value (NPV).

In addition, all enrolled participants were randomly divided into a training set for predictive model construction and a validation set for internal validation of the model performance. To screen the initial risk-associated indicators and avoid overfitting of the multifactorial predictive model, least absolute shrinkage and selection operator (LASSO) regression analysis was performed. The optimal λ value in LASSO regression was determined via the cross-validation method. On this basis, univariate and multivariate logistic regression analyses were further conducted to identify the risk-associated indicators measured at DFU diagnosis for the establishment of a nomogram. The variance inflation factor (VIF) was used to assess multicollinearity among variables in the regression model. The discriminative ability of the constructed nomogram was assessed via the ROC curve, while its calibration degree was verified using calibration plots and the Hosmer–Lemeshow test. Decision curve analysis (DCA) was also adopted to evaluate the clinical utility and net clinical benefit of the nomogram in DFU risk prediction.

All statistical analyses were completed using R software (Version 4.5.1) and EmpowerStats software (Version 2.0). A two-tailed P value < 0.05 was defined as statistical significance for all tests.

## 3 Results

### 3.1 Baseline characteristics of participants

In accordance with the predefined inclusion and exclusion criteria, a total of 1,002 patients were ultimately enrolled in the present study. Intergroup comparisons revealed that the prevalence of PVD and PN was markedly higher in the DFU group than in the non-DFU group (66.1% vs. 26.1%, *p* < 0.001; 80.5% vs. 37.8%, *p* < 0.001, respectively). Meanwhile, the DFU group presented with significantly lower levels of lymphocyte count, hemoglobin and albumin compared with the non-DFU group (2.0 ± 0.7 vs. 2.1 ± 0.7, *p* < 0.001; 133.3 ± 18.6 vs. 138.2 ± 14.8, *p* < 0.001; 38.7 ± 5.2 vs. 41.4 ± 3.6, *p* < 0.001, respectively). Analyses of other clinical and laboratory variables further identified notable intergroup differences: patients in the DFU group were on average older (62.2 ± 8.1 vs. 56.8 ± 11.8, *p* < 0.001) and had a higher proportion of male subjects (66.5% vs. 51.5%, *p* < 0.001) and smokers (43.0% vs. 26.4%, *p* < 0.001). In terms of laboratory indices, the DFU group exhibited significantly elevated levels of glycosylated hemoglobin, neutrophil percentage, white blood cell count and NPAR relative to the non-DFU group (8.8 ± 1.8 vs. 8.4 ± 1.7, *p* = 0.002; 63.0 ± 11.8 vs. 58.0 ± 11.3, *p* < 0.001; 9.5 ± 3.7 vs. 7.1 ± 1.9, *p* < 0.001; 16.7 ± 4.3 vs. 14.1 ± 3.0, *p* < 0.001, respectively). Additionally, the distribution of BMI categories differed significantly between the two groups, with the DFU group having a higher proportion of underweight patients (BMI < 20 kg/m², 31.1% vs. 18.1%, *p* < 0.001) and overweight patients (BMI ≥ 25 kg/m², 48.6% vs. 45.4%, *p* < 0.001) (**Table 1**).

**Table 1:** Characteristics of the study population.

Variables	Non-DFU (N=751, 75.0%)	DFU (N=251, 25.0%)	*P* value
Age (year, mean ± SD)	56.8 ± 11.8	62.2 ± 8.1	<0.001
Sex (%)			<0.001
Male	51.5%	66.5%	
Female	48.5%	33.5%	
BMI (kg/m², %)			<0.001
<20	18.1%	31.1%	
≥20, <25	36.5%	20.3%	
≥25	45.4%	48.6%	
Residential area (%)			0.796
Urban area	53.9%	53.0%	
Rural area	46.1%	47.0%	
Smoke (%)			<0.001
No	73.6%	57.0%	
Yes	26.4%	43.0%	
Alcohol consumption status (%)			0.785
No	74.4%	75.3%	
Yes	25.6%	24.7%	
Hypertension (%)			0.328
No	61.7%	58.2%	
Yes	38.3%	41.8%	
CVD (%)			0.824
No	69.4%)	70.1%	
Yes	30.6%)	29.9%	
PVD (%)			<0.001
No	73.9%	33.9%	
Yes	26.1%	66.1%	
PN (%)			<0.001
No	62.2%	19.5%	
Yes	37.8%	80.5%	
Hemoglobin (g/L, mean ± SD)	138.2 ± 14.8	133.3 ± 18.6	<0.001
Glycosylated hemoglobin (%, mean ± SD)	8.4 ± 1.7	8.8 ± 1.8	0.002
Fasting blood glucose (mmol/L, mean ± SD)	10.0 ± 4.0	10.4 ± 4.8	0.174
Albumin (g/L, mean ± SD)	41.4 ± 3.6	38.7 ± 5.2	<0.001
Lymphocyte (10^9^/L, mean ± SD)	2.1 ± 0.7	2.0 ± 0.7	0.009
Neutrophil percentage (%)	58.0 ± 11.3	63.0 ± 11.8	<0.001
White blood cell (10^9^/L, mean ± SD)	7.1 ± 1.9	9.5 ± 3.7	<0.001
Uric acid (μmol/L, mean ± SD)	310.4 ± 89.7	310.1 ± 92.8	0.961
Creatinine (μmol/L, mean ± SD)	79.6 ± 69.4	76.2 ± 83.1	0.516
eGFR (mL/(min · 1.73 m^2^), mean ± SD)	94.1 ± 20.2	94.8 ± 12.0	0.638
NPAR (mean ± SD)	14.1 ± 3.0	16.7 ± 4.3	<0.001

DFU, diabetic foot ulcer; NPAR, neutrophil percentage to albumin ratio; CVD, cardiovascular disease; BMI, body mass index; eGFR, estimated glomerular filtration rate; PVD, peripheral vascular disease; PN, peripheral neuropathy; SD, standard deviation; %, weighted percentage.

### 3.2 Correlation between NPAR and DFU

#### 3.2.1 Total analyses

**Figure 1:**
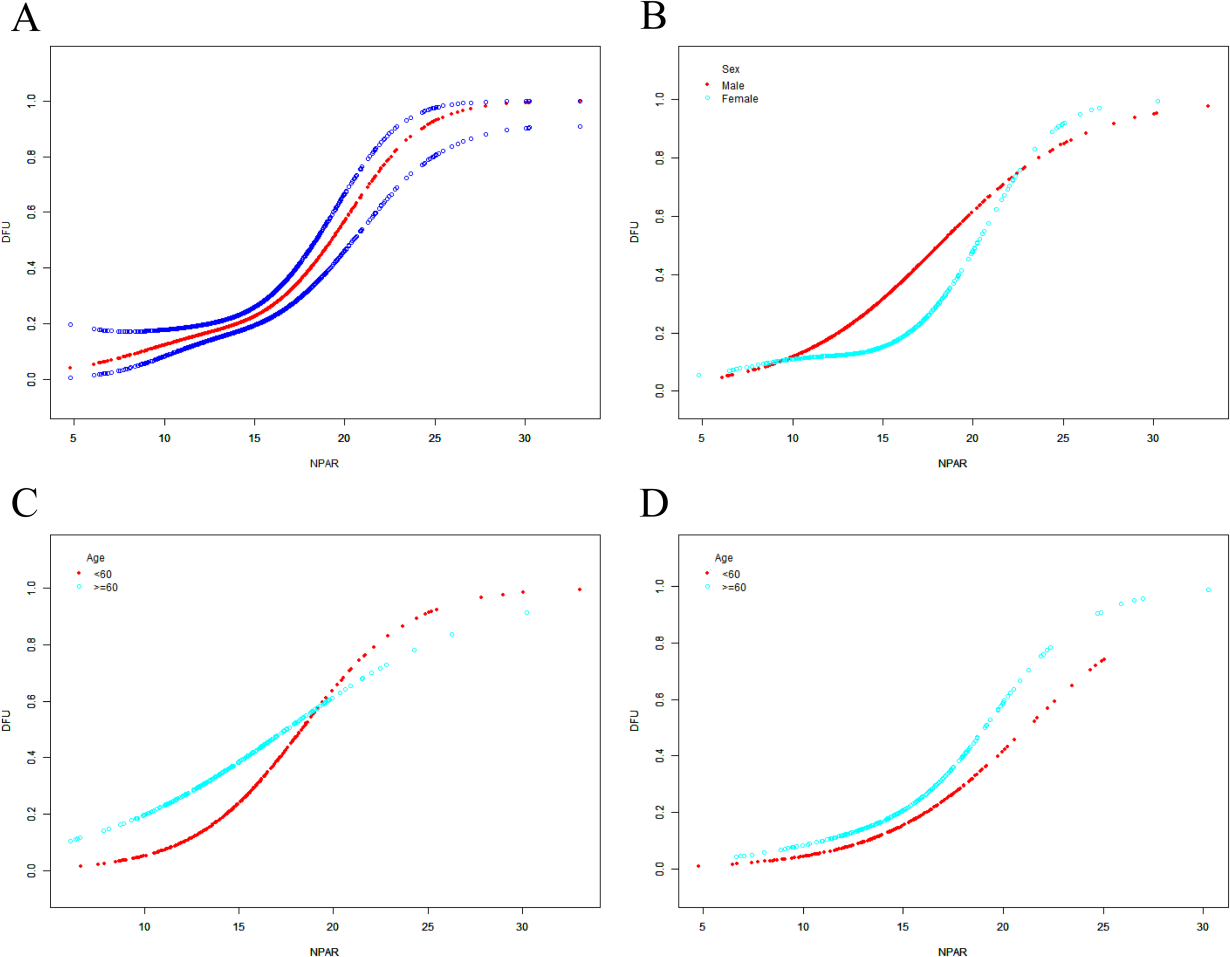
The SCF for the associations of NPAR with the risk of DFU (The sample size of n=1002). [**(A)**, all participants] Participants were not grouped. [**(B)**, all participants] Participants were grouped by sex. [**(C)**, Male; **(D)**, Female] Participants were cross-grouped by age and sex. Age [in **(A, B)**], sex [in **(A)**], BMI, residential area, smoke, alcohol consumption status, hypertension, CVD, PVD, PN, hemoglobin, glycosylated hemoglobin, fasting blood glucose, lymphocyte count, uric acid, creatinine, and eGFR were adjusted. SCF, smooth curve fitting; DFU, diabetic foot ulcer; NPAR, neutrophil percentage to albumin ratio; CVD, cardiovascular disease; BMI, body mass index; eGFR, estimated glomerular filtration rate; PVD, peripheral vascular disease; PN, peripheral neuropathy. The vertical axis represented the probability of DFU.

Logistic regression analyses demonstrated a significant positive correlation between NPAR and DFU risk in the unadjusted crude model (Model 1). Specifically, each 1-unit increment in NPAR was linked to a notable elevation in DFU risk, with an OR of 1.254 (95% CI: 1.195–1.315). This significant positive association remained consistent following stepwise covariate adjustment: in Model 2, after adjusting for age, sex and BMI, the association remained significant (OR = 1.252, 95% CI: 1.193–1.315); in the fully adjusted Model 3 with all covariates incorporated, the positive correlation was still evident and showed a strengthened trend (OR = 1.293, 95% CI: 1.212–1.378) (**Table 2**). When NPAR was stratified into tertile groups for further analysis, compared with the lowest tertile (NPAR.T1) as the reference, the highest tertile of NPAR (NPAR.T3) was associated with a markedly increased DFU risk in the crude analysis, with an OR of 3.536 (95% CI: 2.422–5.162). After adjusting for age, sex and BMI in Model 2, the elevated DFU risk in the NPAR.T3 group relative to NPAR.T1 remained statistically significant (OR = 3.508, 95% CI: 2.391–5.148). Further adjustment for all confounding covariates in Model 3 still confirmed the significant association, with the OR for DFU risk in the NPAR.T3 group reaching 3.706 (95% CI: 2.267–6.058) compared with the NPAR.T1 group (**Table 3**). Notably, linear trend tests revealed a significant linear correlation between increasing NPAR tertiles and the likelihood of DFU diagnosis (P for trend < 0.05, **Table 3**). Consistent with the above findings, SCF combined with GAMs also revealed a similar positive correlation between NPAR and DFU risk, as illustrated in **Figure 1A**.

**Table 2:** Associations of the NPAR and the risk of DFU.

Exposure	Age <60	Age ≥60	Total
Male			
Model 1 β (95% CI) *P* value	1.399 (1.257, 1.556) <0.001	1.146 (1.059, 1.240) 0.001	1.249 (1.175, 1.328) <0.001
Model 2 β (95% CI) *P* value	1.434 (1.278, 1.608) <0.001	1.144 (1.056, 1.239) 0.001	1.249 (1.174, 1.329) <0.001
Model 3 β (95% CI) *P* value	1.514 (1.278, 1.792) <0.001	1.185 (1.049, 1.339) 0.006	1.283 (1.171, 1.406) <0.001
Female			
Model 1 β (95% CI) *P* value	1.357 (1.190, 1.548) <0.001	1.211 (1.104, 1.329) <0.001	1.263 (1.170, 1.364) <0.001
Model 2 β (95% CI) *P* value	1.336 (1.167, 1.530) <0.001	1.209 (1.098, 1.331) <0.001	1.255 (1.160, 1.357) <0.001
Model 3 β (95% CI) *P* value	1.732 (1.217, 2.465) 0.002	1.315 (1.138, 1.520) <0.001	1.340 (1.190, 1.508) <0.001
Total			
Model 1 β (95% CI) *P* value	1.383 (1.273, 1.502) <0.001	1.174 (1.106, 1.247) <0.001	1.254 (1.195, 1.315) <0.001
Model 2 β (95% CI) *P* value	1.398 (1.282, 1.524) <0.001	1.169 (1.100, 1.243) <0.001	1.252 (1.193, 1.315) <0.001
Model 3 β (95% CI) *P* value	1.473 (1.304, 1.663) <0.001	1.217 (1.122, 1.321) <0.001	1.293 (1.212, 1.378) <0.001

Model 1: no covariates were adjusted.

Model 2: age (if applicable), sex (if applicable), and BMI were adjusted.

Model 3: age (if applicable), sex (if applicable), BMI, residential area, smoke, alcohol consumption status, hypertension, CVD, PVD, PN, hemoglobin, glycosylated hemoglobin, fasting blood glucose, lymphocyte count, uric acid, creatinine, and eGFR were adjusted.

DFU, diabetic foot ulcer; NPAR, neutrophil percentage to albumin ratio; CVD, cardiovascular disease; BMI, body mass index; eGFR, estimated glomerular filtration rate; PVD, peripheral vascular disease; PN, peripheral neuropathy; CI, confidence intervals.

**Table 3:** Association of the NPAR.T and the risk of DFU.

Exposure	Age <60	Age ≥60	Total
Male			
Model 1 β (95% CI) *P value*			
NPAR.T			
T1 (4.8-13.2)	1	1	1
T2 (13.2-15.9)	1.732 (0.715, 4.195) 0.223	2.029 (1.105, 3.728) 0.023	1.837 (1.125, 2.999) 0.015
T3 (15.9-33.0)	6.921 (3.104, 15.432) <0.001	2.125 (1.158, 3.899) 0.015	3.537 (2.203, 5.680) <0.001
Model 2 β (95% CI) *P value*			
NPAR.T			
T1 (4.8-13.2)	1	1	1
T2 (13.2-15.9)	1.840 (0.739, 4.585) 0.190	2.003 (1.082, 3.709) 0.027	1.769 (1.079, 2.898) 0.024
T3 (15.9-33.0)	8.968 (3.822, 21.044) <0.001	2.130 (1.157, 3.924) 0.015	3.550 (2.205, 5.715) <0.001
Model 3 β (95% CI) *P value*			
NPAR.T			
T1 (4.8-13.2)	1	1	1
T2 (13.2-15.9)	1.605 (0.484, 5.331) 0.439	3.469 (1.335, 9.018) 0.011	2.104 (1.071, 4.132) 0.031
T3 (15.9-33.0)	7.278 (2.205, 24.023) 0.001	2.566 (1.001, 6.580) 0.039	3.858 (1.961, 7.589) <0.001
*P* for trend	<0.001	0.046	<0.001
Female			
Model 1 β (95% CI) *P value*			
NPAR.T			
T1 (4.8-13.2)	1	1	1
T2 (13.2-15.9)	0.788 (0.202, 3.066) 0.731	1.407 (0.611, 3.240) 0.422	1.217 (0.605, 2.446) 0.582
T3 (15.9-33.0)	5.200 (1.799, 15.028) 0.002	2.680 (1.228, 5.848) 0.013	3.419 (1.825, 6.405) <0.001
Model 2 β (95% CI) *P value*			
NPAR.T			
T1 (4.8-13.2)	1	1	1
T2 (13.2-15.9)	0.797 (0.200, 3.167) 0.747	1.339 (0.566, 3.172) 0.506	1.180 (0.575, 2.421) 0.652
T3 (15.9-33.0)	4.888 (1.644, 14.532) 0.004	2.561 (1.134, 5.785) 0.024	3.266 (1.703, 6.262) <0.001
Model 3 β (95% CI) *P value*			
NPAR.T			
T1 (4.8-13.2)	1	1	1
T2 (13.2-15.9)	0.802 (0.063, 10.214) 0.865	1.166 (0.379, 3.593) 0.789	0.882 (0.337, 2.308) 0.799
T3 (15.9-33.0)	4.845 (2.067, 8.159) 0.015	3.371 (1.094, 10.381) 0.034	4.380 (1.700, 11.289) 0.002
*P* for trend	0.006	0.022	<0.001
Total			
Model 1 β (95% CI) *P value*			
NPAR.T			
T1 (4.8-13.2)	1	1	1
T2 (13.2-15.9)	1.373 (0.660, 2.855) 0.396	1.756 (1.077, 2.863) 0.024	1.597 (1.070, 2.384) 0.022
T3 (15.9-33.0)	6.302 (3.315, 11.979) <0.001	2.378 (1.474, 3.837) <0.001	3.536 (2.422, 5.162) <0.001
Model 2 β (95% CI) *P value*			
NPAR.T			
T1 (4.8-13.2)	1	1	1
T2 (13.2-15.9)	1.443 (0.680, 3.063) 0.339	1.634 (0.996, 2.680) 0.052	1.525 (1.016, 2.290) 0.041
T3 (15.9-33.0)	7.350 (3.756, 14.383) <0.001	2.263 (1.396, 3.669) 0.001	3.508 (2.391, 5.148) <0.001
Model 3 β (95% CI) *P value*			
NPAR.T			
T1 (4.8-13.2)	1	1	1
T2 (13.2-15.9)	1.266 (0.515, 3.110) 0.607	1.640 (0.885, 3.038) 0.116	1.421 (0.871, 2.317) 0.159
T3 (15.9-33.0)	6.348 (2.666, 15.115) <0.001	2.569 (1.368, 4.824) 0.003	3.706 (2.267, 6.058) <0.001
*P* for trend	<0.001	0.003	<0.001

Model 1: no covariates were adjusted.

Model 2: age (if applicable), sex (if applicable), and BMI were adjusted.

Model 3: age (if applicable), sex (if applicable), BMI, residential area, smoke, alcohol consumption status, hypertension, CVD, PVD, PN, hemoglobin, glycosylated hemoglobin, fasting blood glucose, lymphocyte count, uric acid, creatinine, and eGFR were adjusted.

DFU, diabetic foot ulcer; NPAR, neutrophil percentage to albumin ratio; NPAR.T, tertiles of NPAR; CVD, cardiovascular disease; BMI, body mass index; eGFR, estimated glomerular filtration rate; PVD, peripheral vascular disease; PN, peripheral neuropathy; CI, confidence intervals.

#### 3.2.2 Subgroup analyses

Stratified analyses by sex and age both verified a robust positive correlation between NPAR and DFU risk, with the association remaining statistically significant across all stratified subgroups. In male participants, each 1-unit rise in NPAR was linked to a notable increase in DFU risk, yielding an OR of 1.249 (95% CI: 1.175–1.328) in Model 1; this association remained consistent after adjusting for age, sex and BMI in Model 2 (OR = 1.249, 95% CI: 1.174–1.329) and persisted with full covariate adjustment in Model 3 (OR = 1.283, 95% CI: 1.171–1.406) (**Table 2**). When NPAR was stratified into tertiles, the NPAR.T3 was associated with a significantly elevated DFU risk in males relative to the NPAR.T1 in the fully adjusted Model 3, with an OR of 3.858 (95% CI: 1.961–7.589) (**Table 3**). In female participants, a parallel upward trend in DFU risk was observed with increasing NPAR: the ORs for each 1-unit NPAR increment were 1.263 (95% CI: 1.170–1.364) in Model 1, 1.255 (95% CI: 1.160–1.357) in Model 2 and 1.340 (95% CI: 1.190–1.508) in Model 3 (**Table 2**). For female subjects in NPAR.T3, the fully adjusted Model 3 showed a markedly higher DFU risk compared with NPAR.T1, with an OR of 4.380 (95% CI: 1.700–11.289) (**Table 3**).

In accordance with the World Health Organization definition of the elderly, this study adopted 60 years as the cut-off age for age stratification (40, 41). Consistent results were obtained in age-stratified analyses. In participants aged under 60 years, each 1-unit increase in NPAR was associated with a significant rise in DFU risk, with the ORs of 1.383 (95% CI: 1.273–1.502) in Model 1, 1.398 (95% CI: 1.282–1.524) in Model 2 and 1.473 (95% CI: 1.304–1.663) in Model 3 (**Table 2**). The fully adjusted Model 3 further revealed that the NPAR.T3 group had a substantially higher DFU risk than the NPAR.T1 group in this younger subgroup, with an OR of 6.348 (95% CI: 2.666–15.115) (**Table 3**). In participants aged 60 years and above, the positive association between NPAR and DFU risk remained significant, with the ORs for each 1-unit NPAR increment being 1.174 (95% CI: 1.106–1.247) in Model 1, 1.169 (95% CI: 1.100–1.243) in Model 2 and 1.217 (95% CI: 1.122–1.321) in Model 3 (**Table 2**). In this older subgroup, the NPAR.T3 group also exhibited a significantly elevated DFU risk compared with NPAR.T1 in Model 3, with an OR of 2.569 (95% CI: 1.368–4.824) (**Table 3**).

Further cross-stratified analyses by both age and sex confirmed that the positive correlation between NPAR and DFU risk was universally significant across all age-sex subgroups. Specifically, in males under 60 years, each 1-unit NPAR increment was associated with a notable increase in DFU risk, with ORs of 1.399 (95% CI: 1.257–1.556) in Model 1, 1.434 (95% CI: 1.278–1.608) in Model 2 and 1.514 (95% CI: 1.278–1.792) in Model 3 (**Table 2**); the fully adjusted Model 3 showed that NPAR.T3 was linked to a markedly higher DFU risk relative to NPAR.T1 in this subgroup, with an OR of 7.278 (95% CI: 2.205–24.023) (**Table 3**). In males aged 60 years and above, the positive association remained significant, with ORs for each 1-unit NPAR increment being 1.146 (95% CI: 1.059–1.240) in Model 1, 1.144 (95% CI: 1.056–1.239) in Model 2 and 1.185 (95% CI: 1.049–1.339) in Model 3 (**Table 2**); the OR for DFU risk in the NPAR.T3 group compared with NPAR.T1 was 2.566 (95% CI: 1.001–6.580) in the fully adjusted Model 3 (**Table 3**). In females under 60 years, increasing NPAR was also associated with elevated DFU risk: the ORs for each 1-unit NPAR increment were 1.357 (95% CI: 1.190–1.548) in Model 1, 1.336 (95% CI: 1.167–1.530) in Model 2 and 1.732 (95% CI: 1.217–2.465) in Model 3 (**Table 2**), and the NPAR.T3 group had a significantly higher DFU risk than NPAR.T1 in Model 3 (OR = 4.845, 95% CI: 2.067–8.159) (**Table 3**). In females aged 60 years and above, each 1-unit rise in NPAR still correlated with an increased DFU risk, with ORs of 1.211 (95% CI: 1.104–1.329) in Model 1, 1.209 (95% CI: 1.098–1.331) in Model 2 and 1.315 (95% CI: 1.138–1.520) in Model 3 (**Table 2**); the fully adjusted Model 3 indicated that the NPAR.T3 group had a 3.371-fold higher DFU risk compared with the NPAR.T1 group (95% CI: 1.094–10.381) (**Table 3**). Additionally, SCF and GAMs were applied to characterize the potential non-linear relationship between NPAR and DFU risk across the above subgroups, with the results presented in **Figures 1B–D**.

### 3.3 ROC curve

**Figure 2:**
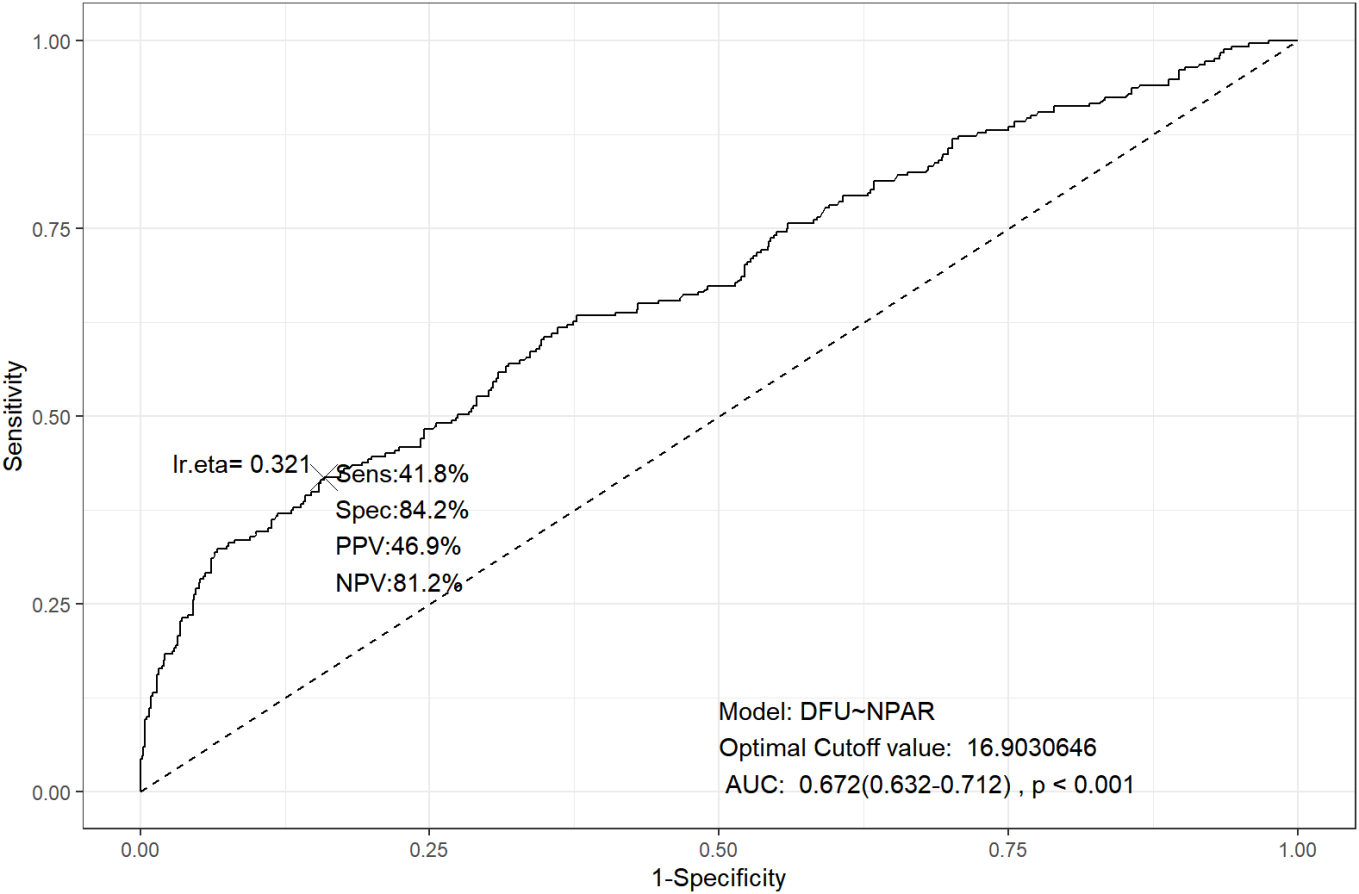
ROC of the NPAR to detect the risk of DFU (The sample size of n=1002). The area under the ROC curve for NPAR in detecting DFU was 0.672 (95% CI: 0.632–0.712). Based on the maximum Youden index, the optimal cut-off value of NPAR for DFU diagnosis was determined to be 16.9, which yielded a diagnostic sensitivity of 41.8% and a specificity of 84.2%. Corresponding to this cut-off value, the corresponding PPV and NPV of NPAR for DFU diagnosis were 46.9% and 81.2%, respectively. ROC, receiver operating characteristic; DFU, diabetic foot ulcer; NPAR, neutrophil percentage to albumin ratio; PPV, positive predictive value; NPV, negative predictive value; CI, confidence intervals.

The diagnostic value of NPAR for DFU was evaluated by means of ROC analysis. As presented in **Figure 2**, the AUC for NPAR in detecting DFU was 0.672 (95% CI: 0.632–0.712). Based on the maximum Youden index, the optimal cut-off value of NPAR for DFU diagnosis was determined to be 16.9, which yielded a diagnostic sensitivity of 41.8% and a specificity of 84.2%. Corresponding to this cut-off value, the corresponding PPV and NPV of NPAR for DFU diagnosis were 46.9% and 81.2%, respectively.

### 3.4 Establishment and validation of the prediction model

**Figure 3:**
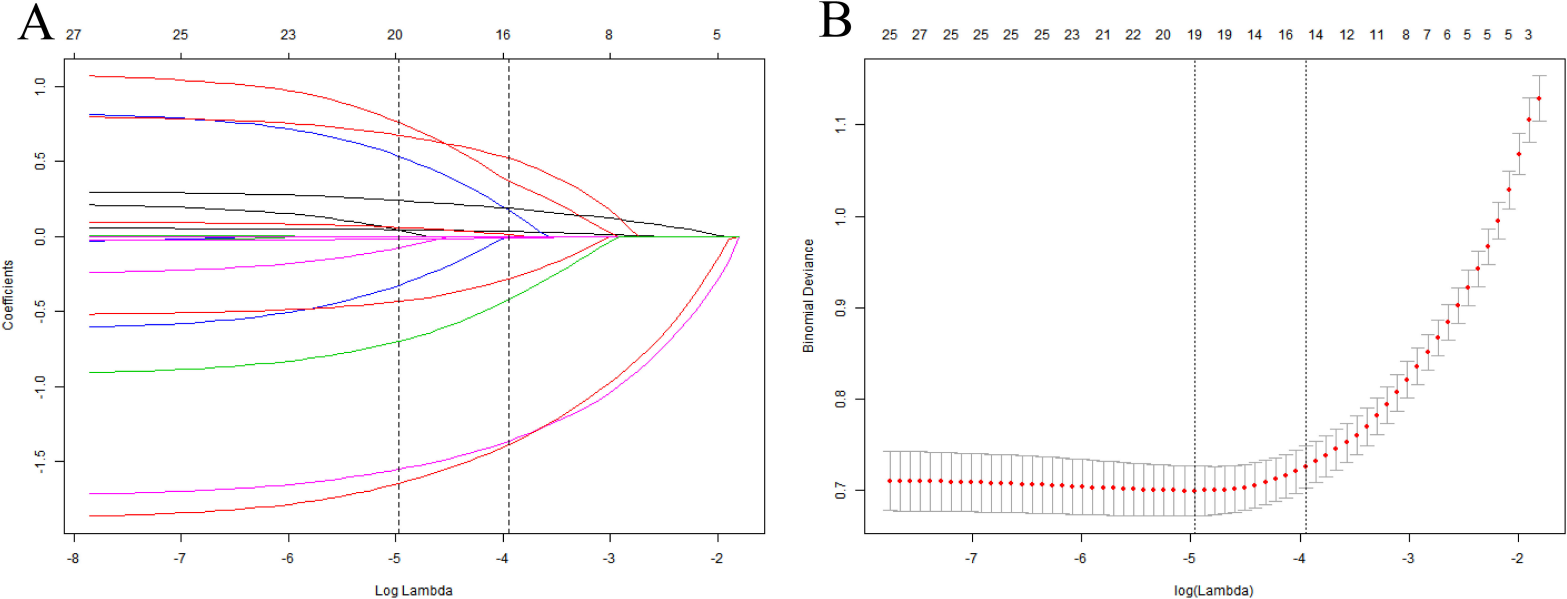
The results of the LASSO regression of the training cohort (The sample size of n=702). **(A)** Each curve in the distribution of regression coefficients represents the trajectory of a specific characteristic coefficient. **(B)** LASSO regression cross-validation profile. The red dot indicates the mean square error corresponding to the λ value, with the vertical axis representing the coefficient values. The number at the top denotes the count of nonzero coefficients in the model, while the horizontal axis shows the logarithmic value of the regularization parameter λ. LASSO, least absolute shrinkage and selection operator.

A total of 1,002 enrolled participants were randomly assigned to a training set (n=700) for predictive model construction and an internal validation set (n=302) for model performance verification. Comparisons of baseline characteristics revealed no significant differences between the two sets in terms of age, sex, BMI, residential area, smoke, alcohol consumption status, CVD, PVD, PN, hypertension, hemoglobin, fasting blood glucose, albumin, lymphocyte count, neutrophil percentage, uric acid, white blood cell count, creatinine, eGFR, NPAR levels and DFU occurrence (all *p*>0.05, **Table 4**). To mitigate the risk of model overfitting, LASSO regression was performed in the training set with DFU as the endpoint, which ultimately identified nine risk-associated indicators measured at diagnosis with non-zero coefficients: age, sex, BMI, smoke, PVD, PN, hemoglobin, glycosylated hemoglobin and NPAR (**Figure 3**). Subsequent multivariate logistic regression analysis further excluded glycosylated hemoglobin, confirmed eight factors were risk-associated indicators measured at DFU diagnosis, with the respective ORs and 95% CIs as follows: NPAR (OR = 1.303, 95% CI: 1.212–1.402), age (OR = 1.058, 95% CI: 1.032–1.083), sex (female vs. male, OR = 0.475, 95% CI: 0.281–0.802), BMI (20–25 kg/m² vs. <20 kg/m², OR = 0.184, 95% CI: 0.094–0.359; ≥25 kg/m² vs. <20 kg/m², OR = 0.445, 95% CI: 0.252–0.788), smoke (yes vs. no, OR = 1.735, 95% CI: 1.023–2.941), PVD (yes vs. no, OR = 5.522, 95% CI: 3.428–8.896), PN (yes vs. no, OR = 6.914, 95% CI: 4.114–11.618), and hemoglobin (OR = 0.981, 95% CI: 0.967–0.996) (**Table 5**). A total of 177 DFU events occurred in the training set, with 8 risk-associated indicators finally included in the model. The calculated event-per-variable was 177/8 ≈ 22, far exceeding the recommended minimum threshold of 10 for clinical predictive models (42). In addition, results of VIF calculations showed no significant multicollinearity among variables in the regression model (**Supplementary Table 1**).

**Table 4:** Distribution of clinical information.

Variables	Training cohort (*N* = 702; 70.1%)	Validation cohort (*N* = 300; 29.9%)	*P* value
Age (year, mean ± SD)	58.0 ± 11.3	58.6 ± 11.0	0.483
Sex (%)			0.767
Male	55.0%	56.0%	
Female	45.0%	44.0%	
BMI (kg/m², %)			0.673
<20	22.1%	19.7%	
≥20, <25	31.9%	33.7%	
≥25	46.0%	46.7%	
Residential area (%)			0.099
Urban area	52.0%	57.7%	
Rural area	48.0%	42.3%	
Smoke (%)			0.721
No	69.8%	68.7%	
Yes	30.2%	31.3%	
Alcohol consumption status (%)			0.056
No	72.9%	78.7%	
Yes	27.1%	21.3%	
Hypertension (%)			0.132
No	59.3%	61.6%	
Yes	40.7%	35.7%	
CVD (%)			0.425
No	68.8%	71.3%	
Yes	31.2%	28.7%	
PVD (%)			0.517
No	66.0%	61.6%	
Yes	34.0%	38.4%	
PN (%)			0.189
No	50.1%	54.7%	
Yes	49.9%	45.3%	
Hemoglobin (g/L, mean ± SD)	136.9 ± 16.2	137.0 ± 15.5	0.906
Glycosylated hemoglobin (%, mean ± SD)	8.4 ± 1.7	8.6 ± 1.8	0.194
Fasting blood glucose (mmol/L, mean ± SD)	10.1 ± 4.0	10.2 ± 4.7	0.699
Albumin (g/L, mean ± SD)	40.6 ± 4.4	41.1 ± 3.9	0.123
Lymphocyte (10^9^/L, mean ± SD)	2.1 ± 0.7	2.0 ± 0.7	0.115
Neutrophil percentage (%)	59.3 ± 11.5	59.2 ± 11.9	0.815
White blood cell (10^9^/L, mean ± SD)	7.7 ± 2.7	7.7 ± 2.8	0.944
Uric acid (μmol/L, mean ± SD)	310.4 ± 90.2	310.2 ± 91.2	0.965
Creatinine (μmol/L, mean ± SD)	80.7 ± 79.8	74.1 ± 53.9	0.191
eGFR (mL/(min · 1.73 m^2^), mean ± SD)	94.7 ± 18.3	93.4 ± 18.9	0.335
NPAR (mean ± SD)	14.8 ± 3.7	14.7 ± 3.2	0.794
DFU (%)			0.855
No	74.8%	75.3%	
Yes	25.2%	24.7%)	

DFU, diabetic foot ulcer; NPAR, neutrophil percentage to albumin ratio; CVD, cardiovascular disease; BMI, body mass index; eGFR, estimated glomerular filtration rate; PVD, peripheral vascular disease; PN, peripheral neuropathy; SD, standard deviation; %, weighted percentage.

**Table 5:** Univariate and multivariate logistic regression for analyzing the DFU associated factors in the training cohort.

Variables	Univariate analysis		Multivariate analysis	
	Odds ratio (95% CI)	*P* ^a^	Odds ratio (95% CI)	*P* ^b^
**Age**	1.049 (1.031, 1.067)	<0.001*	1.058 (1.032, 1.083)	<0.001*
Sex				
Male	1(reference)		1(reference)	
Female	0.521 (0.365, 0.745)	<0.001*	0.475 (0.281, 0.802)	0.005*
BMI				
<20	1(reference)		1(reference)	
≥20, <25	0.299 (0.184, 0.488)	<0.001*	0.184 (0.094, 0.359)	<0.001*
≥25	0.597 (0.397, 0.899)	0.013*	0.445 (0.252, 0.788)	0.005*
Smoke				
No	1(reference)		1(reference)	
Yes	1.949 (1.365, 2.784)	<0.001*	1.735 (1.023, 2.941)	<0.041*
PVD				
No	1(reference)		1(reference)	1(reference)
Yes	5.998 (4.149, 8.673)	<0.001*	5.522 (3.428, 8.896)	<0.001*
PN				
No	1(reference)		1(reference)	
Yes	6.183 (4.107, 9.309)	<0.001*	6.914 (4.114, 11.618)	<0.001*
**Glycosylated hemoglobin**	1.145 (1.044, 1.256)	0.004*	1.110 (0.967, 1.274)	0.139
**Hemoglobin**	0.976 (0.965, 0.987)	<0.001*	0.981 (0.967, 0.996)	0.012*
**NPAR**	1.253 (1.187, 1.323)	<0.001*	1.303 (1.212, 1.402)	<0.001*

*P*^a^, *P* value for odds ratio in univariate logistic regression analysis; *P*^b^, *P* value for odds ratio in Multivariate logistic regression analysis; (*): *P* value < 0.05.

DFU, diabetic foot ulcer; NPAR, neutrophil percentage to albumin ratio; BMI, body mass index; PVD, peripheral vascular disease; PN, peripheral neuropathy; CI, confidence intervals.

**Figure 4:**
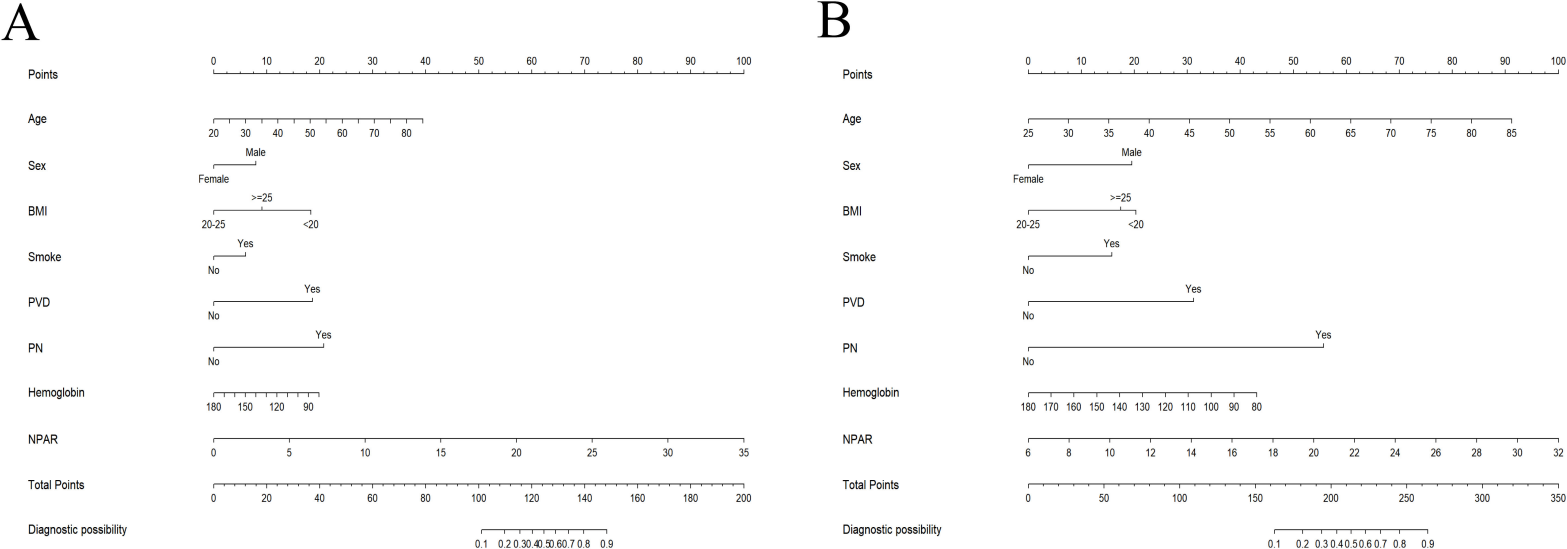
The nomogram for predicting the probability of DFU based on the training [**(A)**, the sample size of n=702] and validation [**(B)**, the sample size of n=300] cohorts. DFU, diabetic foot ulcer; NPAR, neutrophil percentage to albumin ratio; BMI, body mass index; PVD, peripheral vascular disease; PN, peripheral neuropathy.

**Figure 5:**
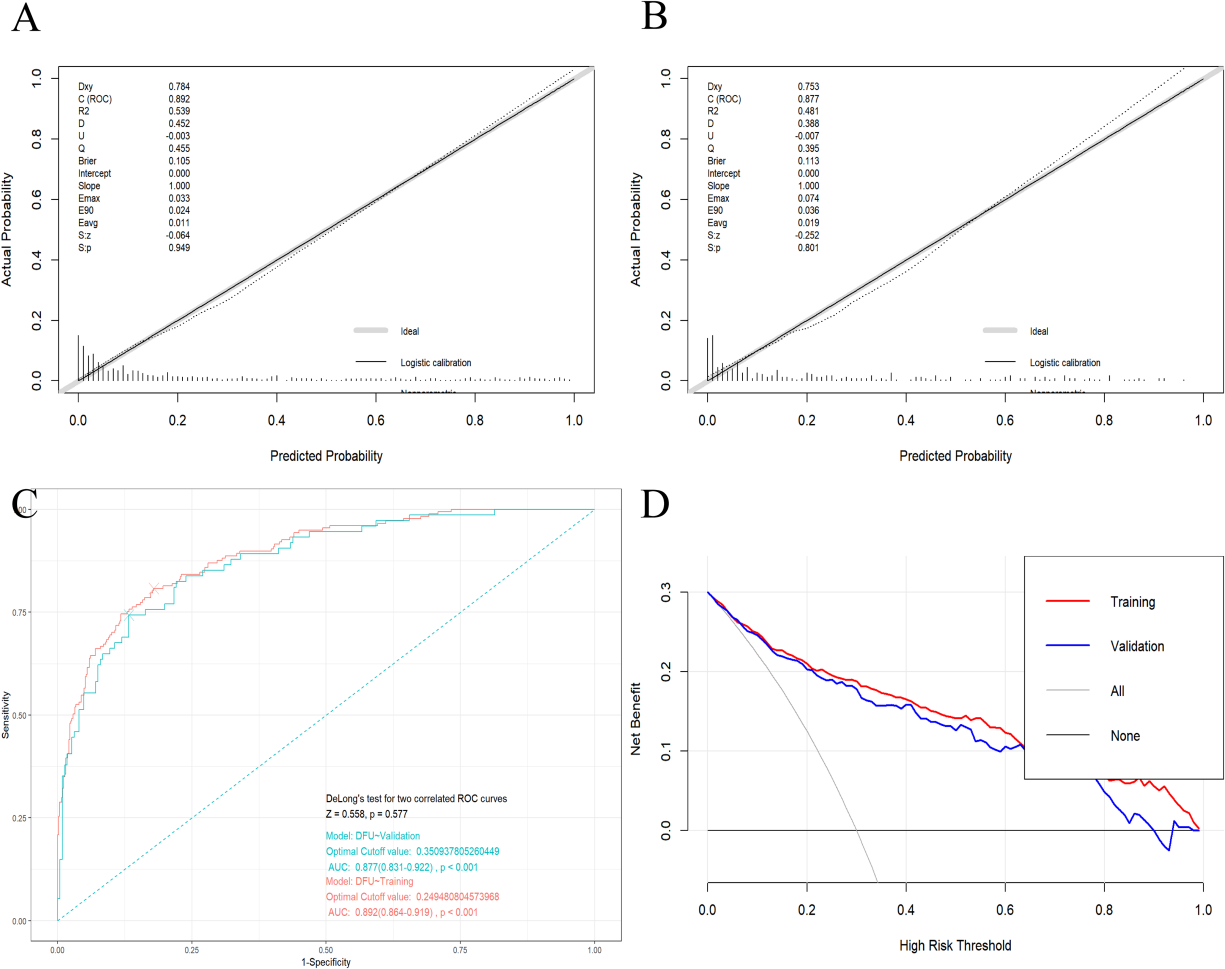
Validation of the prediction model. Calibration curves for the training [**(A)**, the sample size of n=702] and validation [**(B)**, the sample size of n=300] cohorts. **(C)** The ROC curve illustrates the performance of the training (red line) and validation (green line) cohorts of the prediction model. The area under the ROC curve values in the training and validation cohorts were 0.892 (95% CI: 0.864–0.919) and 0.877 (95% CI: 0.831–0.922), respectively, indicating good predictive discrimination. **(D)** The DCA showed that when the nomogram could provide clinical usefulness and net benefit. ROC, receiver operating characteristic; DCA, decision curve analysis; CI, confidence intervals.

A nomogram for individualized DFU risk prediction was established based on the above risk-associated indicators and is visually depicted in **Figure 4**. Calibration curves were plotted to assess the consistency between predicted and actual DFU risk probabilities in the training set (**Figure 5A**) and validation set (**Figure 5B**), respectively; the results exhibited a high degree of agreement in both cohorts, which was further corroborated by non-significant Hosmer–Lemeshow test results (all *p*>0.05), indicating good calibration and fitting of the nomogram model. The discriminative ability of the nomogram was excellent, with the AUC reaching 0.892 (95% CI: 0.864–0.919) in the training set and 0.877 (95% CI: 0.831–0.922) in the validation set. At the optimal cut-off value determined by the maximum Youden index, the predictive model achieved a diagnostic sensitivity of 80.8% and specificity of 82.1% for DFU (**Figure 5C**). Additionally, DCA was conducted to evaluate the clinical utility of the nomogram, and the results showed that the model yielded a higher net clinical benefit than the extreme strategies of “treating all patients” and “treating no patients” across a broad range of threshold probabilities for DFU risk (**Figure 5D**).

## 4 Discussion

The present research verified a notable positive association between the NPAR and DFU in Chinese individuals with diabetes. Subgroup analyses stratified by age and sex further validated the stability and robustness of this association, highlighting its consistency across different demographic subgroups. Findings from both LASSO and logistic regression analyses revealed that increased NPAR serves as a risk-associated indicator measured at diagnosis of DFU. Guided by these results, we developed a DFU predictive model by integrating NPAR with other well-established risk-associated indicators and subsequently conducted internal validation. Our data emphasize the critical link between combined nutritional and inflammatory status (as reflected by NPAR) and DFU development. To our knowledge, this is the first study exploring the potential interplay between NPAR and DFU risk. More importantly, it innovatively incorporates a composite nutritional-inflammatory marker into DFU-related predictive models, thus providing novel perspectives for the early screening and proactive prevention of DFU in clinical practice.

In the pathological progression of DFU, malnutrition and chronic inflammation are not discrete pathological entities but a tightly interwoven comorbidity with a bidirectional causal relationship (43–45). Neutrophils exert a dualistic role throughout the entire course of DFU onset and progression. While they act as the primary line of defense against invasive wound infections, their functional dysregulation and excessive activation emerge as key pathological factors that aggravate local tissue injury, and impede ulcer healing (46–48). In patients with DFU, pathological factors such as persistent inflammation, hyperglycemia and local hypoxia induce delayed apoptosis and excessive activation of neutrophils, leading to the excessive generation of reactive oxygen species (ROS) and neutrophil extracellular traps (NETs). Excessive ROS production impairs the structural and functional integrity of vascular endothelial cells, fibroblasts and epithelial cells at the ulcer site, exacerbating local oxidative stress injury. Although NETs exert a pathogen-trapping effect, their excessive formation and deposition in the wound bed degrade key extracellular matrix components such as collagen and fibronectin, and simultaneously inhibit fibroblast proliferation and collagen synthesis. This directly compromises the tissue integrity required for wound healing, potentially rendering DFU refractory to conventional treatment (46–48).

Furthermore, systemic chronic inflammation triggered by neutrophil hyperactivation shifts hepatic protein synthesis priorities toward acute-phase reactants such as CRP, while suppressing the synthesis of nutritional proteins including albumin and prealbumin (49–51). At the same time, it activates systemic catabolic pathways, leading to accelerated breakdown of skeletal muscle proteins and consequent malnutrition and amyotrophy in DFU patients (52). Lower limb amyotrophy exacerbates pre-existing foot deformities and further disrupts plantar pressure distribution. This mechanical aberration not only increases the susceptibility to new pressure ulcers but also hinders the healing of existing lesions (53). In parallel, malnutrition, particularly characterized by deficiencies in protein and micronutrients, can suppress the chemotaxis, phagocytosis and bactericidal activity of macrophages and neutrophils, and reduce lymphocyte proliferative potential as well as the production efficiency of specific antibodies (54, 55). This comprehensive immune dysfunction may drastically impairs the body’s ability to clear invading pathogens, ultimately contributing to a vicious pathological cycle in DFU (44, 56).

Currently, clinical practice lacks specific biomarkers that can accurately and comprehensively evaluate the systemic inflammatory status and nutritional condition of DFU patients. Conventional single-marker indicators such as CRP and albumin are insufficient to meet the clinical demand for comprehensive patient assessment due to their inherent limitations in sensitivity and specificity (57). However, as a composite biomarker integrating neutrophil percentage and serum albumin levels, NPAR may offer a more integrated reflection of the body’s nutritional status and the degree of systemic inflammation, thereby potentially addressing this clinical gap to a certain extent. The observed positive correlation between NPAR and DFU risk is consistent with the biological rationale underlying this marker, as elevated NPAR often coincides with an impaired inflammatory-nutritional status, which is a recognized risk-associated indicator for DFU pathogenesis. However, the AUC for NPAR alone in detecting DFU was 0.672 (95% CI: 0.632–0.712), which indeed indicates that its standalone diagnostic efficacy is limited, consistent with the inherent limitations of a single biomarker. Therefore, in this study, we further integrated NPAR with clinical factors, including age, sex, BMI, smoke, PVD, PN, and hemoglobin to develop a predictive model. The AUC of the model increased to 0.892 (95% CI: 0.864–0.919), with sensitivity and specificity reaching 80.8% and 82.1%, respectively. This model outperformed the single indicator significantly and had greater clinical application value. Notably, a well-supported causal relationship is recognized between DFU and PVD as well as PN, two well-recognized core risk factors for diabetic foot pathology (10). Findings from a large-scale cohort study have shown that approximately 50% of patients with diabetes develop PN within 25 years of diagnosis, and more critically, PN is implicated in up to 85%-90% of hospitalized DFU cases (58, 59). Sensory neuropathy causes loss of protective sensation, predisposing patients to occult minor foot injuries; motor neuropathy induces foot deformities and biomechanical disturbances, altering plantar pressure distribution and resulting in pressure-related skin breakdown (58, 59). Autonomic neuropathy reduces perspiration, causing dry, fragile skin with impaired barrier function and elevated infection risk, while exacerbating local hypoxia via microvascular dysfunction (60). In addition, PVD contributes to the development of foot ulcers in nearly 50%-70% of diabetic patients. The resulting lower limb ischemia and insufficient nutrient/oxygen supply further increase foot ulcer vulnerability (10).

For elderly diabetic patients, in addition to age-related physiological degeneration and the cumulative burden of comorbidities, long-term neurovascular damage, metabolic disorders, and malnutrition are more prevalent, coupled with relatively poor self-management capacity, collectively elevating their DFU risk (61, 62). Notably, the incidence of DFU in male diabetic patients is approximately 1.5-fold higher than that in females, a disparity attributable to multiple sex-related factors (63, 64). Firstly, female patients generally exhibit better compliance with health management measures, daily self-care practices, and routine foot self-screening compared with male counterparts (65). Secondly, the smoking rate is considerably higher among male diabetic patients, and smoking is a well-established high-risk factor for PVD, PN, and impaired wound healing, with a significant dose-dependent positive correlation with DFU occurrence. Cigarette components including nicotine, carbon monoxide, and cyanide exert deleterious effects through multiple pathological mechanisms: stimulating vascular vasoconstriction to exacerbate tissue ischemia, inducing neuronal damage, suppressing immune function, and exerting direct cytotoxicity on somatic cells. Moreover, reduced hemoglobin levels exacerbate systemic and local tissue hypoxia, which fundamentally impairs the cellular energy metabolism required for normal wound healing (66, 67). Conversely, DFU itself can induce anemia via persistent chronic inflammation and malnutrition, and subgroup analyses across different patient cohorts and analytical models have consistently confirmed a significant association between anemia and elevated DFU risk (68). Furthermore, relevant clinical studies have suggested a non-linear association between BMI and DFU risk, with both obesity and frailty emerging as distinct potential risk factors for DFU (69–71). Obesity exacerbates systemic chronic low-grade inflammation and insulin resistance, thereby accelerating vascular endothelial damage and atherosclerotic progression; additionally, overweight status directly increases peak plantar pressure several-fold, which is the primary mechanical trigger for neuropathic foot ulcers (69). In contrast, frailty, characterized by progressive malnutrition and reduced physical function, is significantly associated with DFU onset, as well as increased risks of lower extremity amputation and all-cause mortality in DFU patients (70, 71).

Based on the above risk factors, this study developed a DFU predictive model and visualized the disease onset risk via a nomogram. The AUC of the training cohort reached 0.892 (95% CI: 0.864-0.919), with the model achieving a diagnostic sensitivity of 80.8% and specificity of 82.1% for DFU, exhibiting excellent discriminative capability. As is well established, DFU has a complex pathogenesis involving multiple pivotal factors, and the weight, combination and interaction modes of these factors vary remarkably among individual patients, rendering the early prediction of DFU highly challenging (30–33). Nevertheless, the nomogram is able to translate complex statistical models into an intuitive graphical scoring system, allowing clinicians to rapidly and individually assess the probability of DFU occurrence in specific patients. This tool therefore holds potential for addressing the complex challenge of DFU risk prediction and may be promising in guiding clinical decision-making (72). In this nomogram, each variable is assigned a specific score that reflects its respective contribution to DFU risk, and a higher total score indicates an elevated disease risk. For patients identified as high-risk, clinicians should maintain a high degree of vigilance and implement targeted early preventive measures for DFU, including standardized foot care, regular self-screening and personalized health management interventions. The DFU predictive model established in this study, which integrates NPAR with other clinical characteristics, may assist clinicians in optimizing early diagnostic strategies and formulating individualized treatment regimens for diabetic patients, and may also enable timely early risk warning for patients and their families, thereby potentially contributing to the reduction of subsequent DFU incidence in the diabetic population.

This study possesses several notable strengths. First, it represents the maiden attempt to introduce NPAR as a novel risk-associated indicator measured at DFU diagnosis, while conducting an in-depth investigation into the correlation between these two factors. Following confirmation of the association between NPAR and DFU, we further developed a clinical prediction model and validated its efficacy and potential clinical utility. Second, the analytical process comprehensively accounted for potential confounding variables, and stratified subgroup analyses by age and sex were performed to enhance the robustness and generalizability of the study findings. Nevertheless, this research is not without limitations. To begin with, although the adoption of consecutive sampling ensured the objectivity of the study cohort to a certain degree, the retrospective nature of this research inherently constrained the strength of evidence supporting causal inferences. This study was also limited by potential selection bias attributed to the single-center setting, as well as compromised enrollment continuity and information bias resulting from missing data. Furthermore, the neutrophil percentage and serum albumin used to calculate NPAR in patients with DFU were obtained at the time of diagnosis, rather than prior to disease onset. Owing to the absence of temporal data documenting the progression from diabetes to DFU, the evidence supporting the longitudinal risk prediction of DFU may be inadequate. Therefore, further prospective studies conducted before the onset of DFU are warranted to validate the conclusions drawn herein. Second, despite adjusting for multiple covariates in the statistical analyses, residual confounding arising from unmeasured factors, such as diabetes duration, use of glucose-lowering medications, and nutritional supplement intake, cannot be entirely ruled out. Furthermore, we did not conduct further stratified analyses on the association between NPAR and different types and severity grades of DFUs. Further relevant studies are warranted to expand this research. Finally, the predictive model was only internally validated using data from a single center and lacked external validation. The optimal cut-off value of NPAR identified in this study was based on data from the Chinese population. Due to factors such as regional differences, ethnic variations, and inconsistencies in laboratory testing standards, this cut-off value may not be applicable to other populations. External validation can address the inherent limitations of internal validation methods by testing the model in an independent external cohort, thereby mitigating overfitting bias and ensuring its applicability across diverse clinical populations, including different medical centers, patient subgroups, and diagnostic criteria. Therefore, future external validation using multi-center datasets will be essential to further enhance the scientific rigor of the model and its applicability in a broader range of clinical settings.

## 5 Conclusion

In summary, the findings of this study reveal that higher NPAR levels correlate with an elevated risk of DFU in the Chinese diabetic population, which reflects a positive association between DFU occurrence and the integrated nutritional-inflammatory status quantified by NPAR. The DFU predictive model integrated with NPAR was validated to have favorable efficacy and clinical applicability, thus providing robust evidence for the potential of NPAR as a novel risk-associated indicator measured at DFU diagnosis. Nonetheless, further large-scale prospective investigations are required to verify the potential causal relationships underlying the observed associations in this study.

## Data Availability

The raw data supporting the conclusions of this article will be made available by the authors, without undue reservation.
