# Supplementary material for: Establishment and evaluation of a novel tool based on inflammation-nutrition derived biomarkers for early diagnosis of diabetic foot ulcers
Source: Front Immunol. 2026 Mar 17;17:1794011. doi: 10.3389/fimmu.2026.1794011 (PMC13035517; doi:10.3389/fimmu.2026.1794011)
Supplement: Supplementary file 1 [file Table1.docx]

Supplementary Table 1

|  | GVIF | Df | GVIF^(1/(2*Df)) |
| --- | --- | --- | --- |
| Age | 1.022556 | 1 | 1.011215 |
| Sex | 1.235969 | 1 | 1.111742 |
| BMI | 1.023314 | 2 | 1.005778 |
| Smoke | 1.230290 | 1 | 1.109184 |
| PVD | 1.086573 | 1 | 1.042388 |
| PN | 1.053838 | 1 | 1.026566 |
| NPAR | 1.062575 | 1 | 1.030813 |

NPAR, neutrophil percentage to albumin ratio; BMI, body mass index; PVD, peripheral vascular disease; PN, peripheral neuropathy; GVIF, generalized variance inflation factor; Df, degree of freedom
